# Inflammation Induced by Perfluorocarbon Liquid: Intra- and Postoperative Use

**DOI:** 10.1155/2014/907816

**Published:** 2014-03-24

**Authors:** Marta S. Figueroa, Diego Ruiz Casas

**Affiliations:** ^1^Hospital Universitario Ramón y Cajal, Madrid, Spain; ^2^Vissum Madrid, Madrid, Spain

## Abstract

Perfluorocarbon liquids (PFCLs) are useful and safe surgical tools in vitreoretinal surgery. The use of PFCL as a tamponade has been controversial due to the corneal toxicity, retinal infiltration, and inflammatory reaction in experimental studies. Several authors have studied in humans the anatomical and functional outcome and adverse effects of perfluorocarbon liquids used as short-, medium-, and long-term tamponade. PFCLs develop dispersion a few days after injection and droplets may move into the anterior chamber and cause corneal endothelial damage. When PFCLs are used as postoperative tamponades for more than one week, a foreign-body inflammatory reaction is observed in up to 30% of cases but such a reaction does not induce PVR, and it resolves after removal of PFCLs. Although most clinical studies have found no signs of retinal toxicity such as progressive visual acuity deterioration or macular anatomical changes, few performed ERG or retinal histological analysis.

## 1. Perfluorocarbon Liquids 

Perfluorocarbon liquids (PFCLs) were introduced by Chang in 1987 as a tool to manipulate the retina in retinal detachment (RD) surgery. Since their first use in humans, PFCLs have improved retinal reattachment rates in RD surgery and increased their uses in vitreoretinal surgery [1].

PFCLs are synthetic fluorinated hydrocarbons fluids that are odorless and colorless, having low viscosity, and heavier than water. These features make PFCLs extremely useful tools in vitreoretinal surgery. Their optical clarity and refractive index allow surgical maneuvers under a visible PFCL-fluid interface. Their weight flattens the retina from posterior to anterior whilst draining the subretinal fluid. Their high interfacial tension keeps the PFCL bubble as a single bubble. Their low viscosity allows easy injection and aspiration and their high boiling point allows for endophotocoagulation under PFCL.

There are several PFCLs that have been studied for vitreoretinal surgery use; see Table 1 [2].

PFCLs are used mainly as intraoperative tools for the following purposes: to flatten the retina in RD, to peel membranes in proliferative vitreoretinopathy (PVR), to shave the vitreous base, to reattach giant retinal tears (GRT), to protect the macular area or lift dropped lenses, to drain suprachoroidal hemorrhage, to stop bleeding, to dissect membranes in proliferative diabetic retinopathy, or to peel the internal limiting membrane [2–5].

PFCLs have even been used as perfusion fluid for the complete vitrectomy procedure in complex retinal detachment cases due to proliferative diabetic retinopathy, rhegmatogenous RD, or vitreous biopsy procedures [6, 7].

The use of PFCL, as a vitreoretinal intraoperative tool, even with high volumes, does not appear to induce any inflammatory reaction or iatrogenic damage, as it has a good safety profile. However, toxicity from extended intraocular use has been reported in animals and humans when PFCLs are retained for more than 48 hours. This toxicity causes an inflammatory response, and it is generally agreed that PFCL should be removed at the end of surgery. This chapter will summarize the current state of knowledge of the intraoperative and postoperative use of PFCLs [8, 9].

## 2. Experimental Studies of PFCL in Animals

PFCLs in the anterior chamber showed inflammatory reaction and corneal damage.

When half of the anterior chamber was filled with PFCLs (perfluorodecalin or perfluorophenantrene) the rabbit eye showed severe inflammation, mainly around the lower limbus in the first postoperative days. Within a week, the rabbit eyes developed corneal haziness due to stromal edema. This edema affected the whole corneal area, and not only the inferior half of the cornea, in two-thirds of the specimens. The corneal edema decreased after 2-3 weeks, and small clusters of exudates on the surface of the PFCL droplets could be seen. PFCL was removed after 2 or 4 weeks later, most of the specimens developed corneal scarring, particularly at the margin of the droplets. In addition, half of the specimens developed subepithelial vessel ingrowth [10].

Histological analysis found stromal edema, irregularly thickened endothelium, and vacuoles in the endothelial cells, iris, and inferior trabecular meshwork. In a few eyes a small number of macrophages were observed in the iris. The endothelial cell count persistently decreased by 50%, scars with fibroblast ingrowth formed, and subepithelial neovascular vessels developed inferiorly, whereas a monolayer of endothelial cells was observed superiorly. Chamber angle synechia was observed in the inferior angle [10–12].

If a minimal amount of PFCL was present in the anterior chamber, there was no corneal decompensation. Moreover, corneal thickness, endothelial cell density, and morphology remained unchanged. However, the histological analysis showed exudates in the inferior chamber angle and vacuoles in the inferior trabecular meshwork 8 weeks after injection [13].

Thus, the effects of PFCL in the anterior chamber depend on the amount of PFCL. When there is a high volume, endothelial cell damage occurs quickly as PFCL blocks endothelial cell nutrition. This leads to corneal decompensation and fibrotic tissue begins to replace the endothelium 2 weeks after anterior chamber injection [10]. Cell damage can be observed after injection of 0.05 mL of PFCL but amounts under 0.025 mL appear to induce no reaction in the corneal endothelium, although they cause changes in the trabecular meshwork [11, 12].

The amount of PFCL is reduced by half 2-3 months after injection likely due to absorption through the trabecular meshwork [14].

In one study, the intraocular pressure was not modified by the presence of PFCL, despite the fact that some PFCL dispersion was found [10].

When PFCLs were injected in the vitreous cavity of rabbits after creating space by gas expansion or vitrectomy, PFCL droplets developed a few days after injection but residues were rarely observed in the anterior segment tissues [15, 16].

PFCLs toxicity in the vitreous chamber has been assessed. No significant inflammation was clinically observed during a 4-week follow-up; however, there were histological alterations. PFCLs were observed infiltrating beyond the internal limiting membrane with enlargement of the intercellular spaces among the Müller cells 1 week after the injection in rabbit and pig eyes. The degree of alteration and the number of PFCL droplets increased with longer follow-up. Later, PFCL penetrated deeper through the retinal layers involving the photoreceptor nuclear layer and the outer segment layer and producing morphological changes. The plasma membrane of the retinal cells in contact with PFCLs appeared irreversibly disrupted, and infiltration of the liquid within the retinal discs with cytoplasm degeneration was observed. In the retinal pigment epithelium, PFCL induces alterations within the endogenous lipid-containing bubbles. There was no PFCL found beyond Bruch's membrane [11, 14, 17–22].

The degree of infiltration is related to the viscosity and the tendency of PFCL to emulsify. The histological changes have been observed with all PFCLs: C8F18 in less than 8 to 48 hours, C10F18 in less than 3 hours, C6F13C8H18 in less than 48 hours, and C12F27N in less than 2 days. However, C14F24 seemed to be well tolerated for 6 to 23 weeks [11, 14–22].

There were no ultrastructural changes in the outer plexiform layer and photoreceptors outer segments in rabbit eyes containing PFCL for up to 1 week. However, focal areas of narrowing of the outer plexiform layer and ultrastructural distortion of photoreceptor outer segments were noted in the inferior retina after 2 weeks. These changes could be due to PFCL high specific gravity. Similar changes have been reported in the superior retina of silicone oil-filled eyes [15, 16].

Electroretinogram (ERG) tracings in experimental animals showed alterations in the a and b wave amplitudes during vitreous replacement with PFCL for 48 hours [15].

An inflammatory reaction of monocyte-macrophage cells was observed on the inner surface of the inferior retina after 1 week of PFCL presence in the vitreous cavity. The cytoplasm of these cells appeared to be filled with phagocytosed material, engulfed in lysosomes. However, macrophages did not seem to be organized in epiretinal pseudomembranes. At 4-week follow-up, fibroblast-type cells formed highly organized thick pseudomembranes with a large number of newly formed extracellular matrix components. The inflammatory reaction may be related to the presence of impurities. Further, at one week, IgG, IgM, and complement factor 3 were found in the retina and the choroid, especially around the PFCL droplets. No massive infiltration of cells from the peripheral blood was observed, suggesting that the inflammatory reaction is local [13, 17].

Other authors have also reported deposition of white precipitates at the PFCL-vitreous interface when PFCLs were left in the vitreous cavity for more than 4 weeks. Histopathologic studies identified it as an amorphous proteinaceous material that was acellular, except for macrophages. When animal vitreous and PFCLs are shaken, this white precipitate appears, and it was identified as noncellular denatured proteins consistent with precipitated or compressed vitreous [14, 16, 23].

## 3. Studies of PFCL in Humans

PFCL tamponade in human studies has been arbitrarily classified as short-term (less than 1 week), medium-term (between 1 and 3 weeks), and long-term (more than 3 weeks) tamponade.

### 3.1. PFCL as a Short-Term Intraocular Tamponade

Despite the fact that PFCL is commonly used as an intraoperative tool in vitreoretinal surgery, there is concern about its use as an intraocular vitreous replacement because of the potential histological and electrophysiological changes observed in experimental studies, in addition to reports of potential mechanical compression, submacular migration, and inflammation [9, 24–26]. The origin of PFCL intolerance is not precisely known. It may be a combination of impurities, chemical effects, and mechanical compression. Nevertheless, several studies have used PFCL as short-, medium-, and long-term tamponade. The physical features of PFCLs make them excellent vitreous substitutes for dealing with inferior retinal pathology, where common tamponades with a density lower than water, like silicone oils or gases, are not so effective.

The studies on the use of PFCLs as short-term tamponade are shown in Table 2 [24, 27–29].

When PFCL is used as an intraocular tamponade the reattachment rate reported is high, averaging between 76% and 82% [24, 27–29], which is comparable to the rate obtained with the use of heavy silicone oil [30–32]. The low rate of redetachment when PFCLs are used as a postoperative tamponade may be due to the extended apposition of the retinal tear to the underlying retinal pigment epithelium (RPE), resulting in more effective chorioretinal adhesion. Moreover, the incidence of inferior PVR is reduced because of the lack of pooling of RPE cells, chemoattractants, and serum components on the inferior retina. Redetachment tends to occur in the superior retina because of the lack of tamponade, new superior breaks, or PVR progression [24, 27–29].

VA improvement was observed in 50%–86% of cases [24, 27–29], with no clinical evidence of toxicity, such as a decline in visual acuity during the follow-up, or visible macular changes.

In one case series, an inflammatory reaction was described in 30% of cases. It was associated with hypotony, and it disappeared after PFCL removal [28].

Therefore, the use of PFCL as a short-term tamponade, removing PFCL with or without gas or silicone exchange, did not appear to induce either severe inflammatory reaction or toxic retinal effects (shown by absence of visible macular alterations and recovery of visual acuity) in several clinical reports. However, experimental studies have shown histological infiltration of PFCL droplets through all retinal layers, from the ILM to the RPE, although it is known whether this finding impairs retinal function.

### 3.2. PFCL as a Medium-Term Intraocular Tamponade

When PFCLs were used as a medium-term postoperative tamponade, the primary reattachment rates ranged between 86% and 92% [33–38]. Visual improvement was reported in up to 69% of patients, and the visual acuity results were mainly related to macula status.

The most common causes of retinal redetachment were development of PVR, superior tears, or tears anywhere.

The studies about PFCLs as medium-term tamponade are shown in Table 3 [33–38].

When PFCLs were used for 2 to 3 weeks, a typical granulomatous inflammatory reaction with precipitates was observed on the posterior lens capsule, retina, optic nerve head, or retinal blood vessels in 28% of patients. This reaction was different from the characteristic inflammation observed after vitrectomy, and it appears as white, round, spiculated deposits on the posterior lens surface, within indwelling PFCL and over the retinal surface. The posterior capsule deposits may obscure visualization of the posterior segment [35]. In most instances, vitreous cavity deposits seem to have a perivascular predominance and are more prominent, in the inferior vitreous cavity and retinal surface.

The inflammatory reaction started between 7 and 10 days after surgery, and it progressed in 64% of patients, impairing posterior segment visualization by the time PFCL was removed. Such patients with no foreign-body response within the first 10 days did not develop inflammation later. The inflammation cleared with topical or periocular corticosteroids in all eyes 1 to 3 weeks after PFCL removal. The inflammatory reaction did not correlate with final visual acuity, retinal attachment, PVR development, or persistently high intraocular pressure [33–37].

Histopathologic analysis demonstrated the absence of neutrophils, lymphocytes, or additional inflammatory cells, but rather the presence of numerous macrophages with clear cytoplasmic inclusions consistent with an acute foreign-body-induced phagocytic response. Occasional clumps of extracellular pigment granules were present. The absence of additional inflammatory cells seems to exclude a macrophage response induced by classically activated TH1 (mediated by INF-gamma or TNF-alpha) or traditional alternate TH2 responses. Both responses are associated with inflammatory cell recruitment and the elaboration of extracellular matrix and local tissue destruction; however, PFCL-induced macrophage response was not associated with synechiae, iris atrophy, PVR, retinal toxicity, or any other types of tissue damage [35]. Nevertheless, retinal toxicity was ruled out due to the lack of visual acuity deterioration or visible macular alterations, but it was not evaluated with electrophysiological tests or retinal histology.

One potential source of macrophages is systemic circulation, having migrated from the retina, the ciliary body, or iris vasculature, but the absence of deposits within the anterior chamber indicates that the response may be limited to the vitreous cavity. Another potential source of cells inducing the foreign-body response is residual vitreous macrophages. However, the observed cellular density seems greater than can be accounted for only by this source, especially in the context of recent complete vitrectomy. Central nervous system microglia have shown the ability to locally proliferate through the activity of resident colony-forming cells, which may be the primary source of the macrophage response [35, 39, 40].

Some reports have suggested that the phagocytic response observed within indwelling PFCL is caused by regulatory macrophages. These are distinct macrophage populations that have an inflammation-limiting housekeeping role. Their activity may be enhanced by glucocorticoids, and they produce an anti-inflammatory cytokine, interleukin-10 (IL10). Further cytochemical analysis (IL10 and IL12) may be useful in differentiating the nature of the macrophage population. PFCLs have shown cytoprotective properties, such as the ability to downregulate the toll-like receptor inflammatory pathway (which is essential for lipopolysaccharide-induced cytotoxicity). Therefore, PFCLs may inhibit the macrophage proinflammatory cascade, making glial recurrence of PVR less likely and reducing postoperative inflammation in the early postoperative period [35, 41, 42].

A similar reaction has been described when small amounts of PFCL are left in the eye after PFCL removal. When PFCL accumulated in the retrolental space, between the posterior capsule and the anterior hyaloid, a typical inflammatory reaction appeared. Adjacent to the PFCL debris, there was one layer of flattened epithelial cells (cytokeratin positive, GFAP negative, and melanin positive), which was likely of retinal pigment epithelial origin. Beneath that layer, there was another layer of highly vacuolated cells with brown pigment (CD68 positive) which contained engulfed PFCL. There were no other inflammatory cells. This seems to be a foreign-body reaction induced by altered PFCL. The nature of PFCL can be altered by emulsification, absorption of biological substances, and close tissue contact, and such altered PFCL enhances macrophage phagocytosis. Pigment epithelial cells eventually try to engulf the altered substances, thus causing this typical inflammatory reaction [38].

PFCLs migrate to the anterior chamber in 22% of cases in both phakic and pseudophakic eyes, in the absence of obvious zonular dehiscence [33]. The low viscosity of PFCLs and their high rate of dispersion allow them to course through intact zonules, reach the retroiridal space, and enter the anterior chamber through the pupil.

PFCL in the anterior chamber may block trabecular meshwork outflow, damage the corneal endothelial cells, or induce an inflammatory reaction. When there is a gross presence of PFCL in the anterior chamber, it may induce persistent IOP elevation. The anterior chamber inflammatory reaction was highly correlated with the presence of foreign-body response, indicating that anterior chamber reaction may largely consist of macrophages or that eyes with a severe anterior chamber inflammatory response are more likely to develop foreign-body reaction. However, this inflammatory reaction consists of mild deposits in the angular recesses with no evidence of synechiae [35].

The granulomatous inflammatory reaction is hypothesized to be due to a PFCL induction of local, foreign-body-type, macrophage-stimulating molecular pathway that does not appear to generate structural retinal damage within a 3-year postoperative time period. After PFCL removal, no deposits were observed and no iris synechiae were found. Residual foreign-body deposits appeared as contracted pigmented flecks over the posterior lens capsule and resolved within 1 month after PFCL removal in all cases, rarely leaving residual pigmentation on the posterior lens capsule. Thus, the inflammatory reaction improved after PFCL removal without producing delayed-type hypersensitivity, such as uveitis or sympathetic ophthalmitis or leaving obvious anatomic or visual sequelae.

### 3.3. PFCL as a Long-Term Intraocular Tamponade

The use of long-term PFCL tamponade is a controversial topic due to the experimental observations of outer retinal layer damage in several studies [11, 14, 17–22]. However, PFCLs have been used without clinical evidence of damage to the optic disk or to the retina assessed by the lack of progressive visual deterioration or RPE changes. Retinal reattachment rates ranged between 63% and 90%.

The studies on the use of PFCL as long-term tamponade are shown in Table 4 [9, 43–45].

Although an inflammatory reaction was found in 17% of patients at 2 to 6 weeks after surgery with flare in the anterior chamber and pigment clumps at the back of the lens, the intraocular lens, or the anterior chamber, there was no postoperative PVR.

When a significant quantity of PFCL (more than 0.25 mL) is left in the eye for an extended period of time, an inflammatory reaction develops as early as the third postoperative week in all cases. A white flocculent, flake-like material on various intraocular structures is found on various intraocular structures, such as the posterior lens capsule, the pars plana, the vitreous base, the optic nerve head, and the posterior retina [9, 43]. Histopathologic examination disclosed compression of the residual vitreous, macrophages, and, in some cases, multinucleated giant cells. Macrophages contained intracellular vacuoles filled with electron-lucent material, identified by energy-dispersive spectroscopy as PFCL.

PFCL disperses and migrates in the anterior chamber, inducing corneal edema and endothelial cell loss after 4 weeks of PFCL contact. They may also cause keratic precipitates, deep corneal stromal vessels, and nuclear cataract. Histopathologic examination showed epithelial edema, an extensively deficient Bowman membrane, corneal stroma vascularization with inflammatory cells, and PFCL engulfed in keratocytes and macrophages around the vessels. The endothelium was largely deficient, and a thin collagenous membrane containing melanin pigment was present on the posterior surface of the cornea [46, 47].

## 4. Conclusion

PFCLs are useful and safe intraoperative tools in vitreoretinal surgery that do not induce inflammation. When used as a tamponade, PFCLs achieve excellent anatomical reattachment results, with a primary average success rate of 97–100% under PFCLs and 63–100% after PFCL removal. This outcome may encourage us to accept PFCL as a useful tamponade. However, when PFCLs are used as a postoperative tamponade for more than 1 week, an inflammatory reaction develops in up to 30% of cases in clinical studies, and experimental studies have also shown retinal infiltration by PFCL.

Most clinical studies have not found signs of retinal toxicity such as progressive visual acuity deterioration or macular anatomical changes, but ERG or retinal histological analysis has not been performed.

When PFCLs are left in the vitreous cavity, dispersion develops a few days after injection and PFCL droplets may move into the anterior chamber, although there is no evidence about how much PFCL and how long it should stay in the vitreous cavity to cause this complication. PFCLs in the anterior chamber induce endothelial damage in the long term. Further, PFCLs induce a foreign-body reaction in the vitreous cavity, with macrophages engulfing PFCL droplets. However, this inflammatory reaction does not induce PVR and resolves after PFCLs removal.

Given their adequate physical properties and anatomical results, PFCLs might be a useful vitreoretinal surgery tool to deal with inferior retinal pathology. Nevertheless, retinal toxicity has not been ruled out in humans by means of ERG or histological examination. On the other hand, heavy silicone oil is an approved and safe tool to treat inferior retinal pathology. If PFCL is used as a tamponade, it must be removed completely as soon as possible once the retinopexy is complete, in order to avoid inflammation, dispersion, endothelial damage, or retinal damage. Special care must be taken to avoid using PFCL together with silicone oil or heavy silicone oil, because they can mix generating a new fluid with different physical properties known as sticky silicone oil [48, 49].

## Figures and Tables

**Table 1:** Perfluorocarbon liquids [2].

PFCL	Chemical formula	Molecular weight (g/mol)	Density	Surface tension (Dyn/cm AT 25°C)	Refractive index	Vapor pressure (mmHg AT 37°C)	Viscosity (CST AT 25°C)
Perfluoro-n-octane	C8F18	438	1.76	14	1.27	50	0.8
Perfluorodecalin	C10F18	462	1.94	16	1.31	13.5	2.7
Perfluorophenantrene	C14F24	624	2.03	16	1.33	<1	8.03
Perfluorohexyloctane	C6F13C8H18	433	1.35	20	1.34		2.5

**Table 2:** PFCLs used as short-term tamponade [24, 27–29].

PFCL	Pathology	Tamponade time	Follow-up	Study	Results	Inflammation
C8F18	Inferior RD with PVR	7 days to air, C3F8, or silicone oil	14 months	Case series *N* = 17 (Drury and Bourke 2011) [24]	Primary reattachment after PFCL and tamponade removal 76% VA improvement 65% VA stable 18% Cataract 60% Macular changes 12% Inflammation 6% IOP > 21 29% Retained PFCL 24%	Iris 6 months after PFCL removal

C8F18	RD with giant retinal tear	7–5 days to SF6, C3F8, or silicone oil	24.5 months	Cases series *N* = 62 (Sirimaharaj et al. 2005) [27]	Primary reattachment after PFCL and tamponade removal 80.6% VA improvement 54.8% VA stable 32.3% Cataract 80.5% Macular changes 0% Inflammation 0% Glaucoma 4.8% Retained PFCL 0%	

C8F18	RD with giant retinal tear and PVR	5 days to C3F8 or silicone oil	16 months	Cases series *N* = 10 (Ventura et al. 2007) [28]	Primary reattachment after PFCL and tamponade removal 80% VA improvement 50% VA stable 20% Inflammation 30%	30% hypotony with anterior chamber and vitreous cell reaction

C10F18	RD with GRT and PVR	5 days to fluid	18 months	Cases series *N* = 11 (Bottoni et al. 1994) [29]	Primary reattachment after PFCL removal 82% VA 64% > 20/40 High IOP 30% Inflammation in AC 28% MER 9% ERG normal	28% AC flare or fibrin reaction

**Table 3:** PFCLs used as medium-term tamponade [33–38].

PFCL	Pathology	Tamponade time	Follow-up	Study	Results	Inflammation
C8F18	Inferior RD with or without PVR	17.4 days to SF6	32 months	Case series *N* = 157 (Sigler 2013)	Primary reattachment rate after PFCL and tamponade removal 87.5% Mean VA change in logMAR 0.15 ± 0.87 PFCL in anterior chamber 22% IOP high 34% PFCL in anterior chamber 21% Inflammation 27% Cataract surgery16% Glaucoma surgery 6%	Granulomatous inflammatory precipitates 27%

C8F18	Recurrent inferior RD with PVR	18.3 days to fluid	30.71 months	Case series *N* = 44 (Sigler 2013)	Primary reattachment rate after PFCL removal 86% Mean VA change in logMAR 0.08 ± 0.13 PFCL in anterior chamber 22% IOP high 36% PFCL in anterior chamber 32% Inflammation 32% Cataract surgery 42% Glaucoma surgery 5%	Granulomatous inflammatory precipitates 32%

C8F18	RD with GRT without PVR	16.4 days to C3F8	53.9 weeks	Case series *N* = 16 (Rofail and lee 2005) [36]	Primary reattachment rate after PFCL and tamponade removal 100% Redetachment 6,3% VA improvement 68.8% VA stable 12.5% Cataract 54.5% ERM 25% Hypotony 18.6% Inflammation 6%	Inflammatory reaction in AC after PFCL removal with fibrin over the pupil

C8F18	Inferior RD	19 days to air	29.7 months	Case series *N* = 181 (Sigler 2013)	Primary reattachment rate after PFCL removal 88% Final VA 0.81 ± 0.67 Inflammation 28%	Foreign-body response 28%

C8F18	Inferior RD with or without GRT	11 days		Case series *N* = 39 (Rush et al. 2012) [37]	Primary reattachment rate 92.4% Severe inflammation 20.6% IOP > 21 35.9% Cataract surgery 84%	Mild inflammation 79% Severe inflammation 21% Pupillary membrane 9%

C10F18	RD with GRT	2 weeks to SF6		Single case (Singh et al 2001) [38]	Typical inflammatory reaction 7 days after PFCL removal	Macrophages and epithelial cells

**Table 4:** PFCL as long-term tamponade [9, 43–45].

PFCL	Pathology	Tamponade time	Follow-up	Study	Results	Inflammation
C14H17F13	Inferior RD with or without PVR	76 days to fluid	97 days	Case series *N* = 23 (Kirchhof et al. 2002) [43]	Primary reattachment rate after PFCL removal 78,3% PFCL in anterior chamber 48% IOP high 8,7% by pupil block Inflammation 17% Cataract 90% Dispersion 50% MER 22%	AC flare and pigment cells with pigmented clumps behind lens 17%

C8F18	RD with retained PFCL			Case series *N* = 5 (Elsing et al. 2001) [9]	Inflammatory reaction 100%	White flake-like material of macrophages and multinucleated giant cells

C14F24	RD with GRT	87.2 days to fluid	13.7 months	Case series *N* = 15 (Kertes et al. 1997) [45]	Primary reattachment 63% Cataract 44% PFCL migration 19% High IOP 19% PFCL in anterior chamber 19%	

C14F24	RD	From 5 days to 4 weeks to fluid, SF6, C3F8, or silicone oil	20.32 weeks	Case series *N* = 60 (Verma et al. 1995) [44]	Primary reattachment 90% ERM 7% Residual PFCL 3% Vitreous hemorrhage 2% Choroidal detachment 2% Vitreous fibrinous reaction 4%	Fibrinous reaction in vitreous 4%

## References

[1] Chang S (1987). Low viscosity liquid fluorochemicals in vitreous surgery. *American Journal of Ophthalmology*.

[2] Wong IY, Wong D, Wilkinson CP, Wiedemann P (2013). Special adjunts to treatment. *Retina*.

[3] Arevalo JF (2008). En bloc perfluorodissection for tractional retinal detachment in proliferative diabetic retinopathy. *Ophthalmology*.

[4] Imamura Y, Minami M, Ueki M, Satoh B, Ikeda TM (2003). Use of perfluorocarbon liquid during vitrectomy for severe proliferative diabetic retinopathy. *British Journal of Ophthalmology*.

[5] Brazitikos PD, Androudi S, Dimitrakos SA, Stangos NT (2003). Removal of the internal limiting membrane under perfluorocarbon liquid to treat macular-hole-associated retinal detachment. *American Journal of Ophthalmology*.

[6] Quiroz-Mercado H, Garcia-Aguirre G, Ustáriz-González O, Martín-Avià J, Martinez-Jardon S (2007). Perfluorocarbon-perfused vitrectomy using a transconjunctival 25-gauge system. *Retina*.

[7] Quiroz-Mercado H, Rivera-Sempertegui J, MacKy TA (2005). Performing vitreous biopsy by perfluorocarbon-perfused vitrectomy. *American Journal of Ophthalmology*.

[8] Inoue M, Iriyama A, Kadonosono K, Tamaki Y, Yanagi Y (2009). Effects of perfluorocarbon liquids and silicone oil on human retinal pigment epithelial cells and retinal ganglion cells. *Retina*.

[9] Elsing SH, Fekrat S, Green WR, Chang S, Wajer SD, Haller JA (2001). Clinicopathologic findings in eyes with retained perfluoro-n-octane liquid. *Ophthalmology*.

[10] Stolba U, Krepler K, Velikay M, Binder S (1999). Anterior segment changes in rabbits after experimental aqueous replacement with various amounts of different perfluorocarbon liquids. *Graefe’s Archive for Clinical and Experimental Ophthalmology*.

[11] Nabih M, Peyman GA, Clark LC (1989). Experimental evaluation of perfluorophenanthrene as a high specific gravity vitreous substitute: a preliminary report. *Ophthalmic Surgery*.

[12] Moreira H, de Queiroz JM, Liggett PE, McDonnell PJ (1992). Corneal toxicity study of two perfluorocarbon liquids in rabbit eyes. *Cornea*.

[13] Sparrow JR, Ortiz R, MacLeish PR, Chang S (1990). Fibroblast behavior at aqueous interfaces with perfluorocarbon, silicone, and fluorosilicone liquids. *Investigative Ophthalmology and Visual Science*.

[14] Chang S, Zimmerman NJ, Iwamoto T, Ortiz R, Faris D (1987). Experimental vitreous replacement with perfluorotributylamine. *American Journal of Ophthalmology*.

[15] Chang S, Sparrow JR, Iwamoto T, Gershbein A, Ross R, Ortiz R (1991). Experimental studies of tolerance to intravitreal perfluoro-n-octane liquid. *Retina*.

[16] Eckardt C, Nicolai U, Winter M, Knop E (1991). Experimental intraocular tolerance to liquid perfluorooctane and perfluoropolyether. *Retina*.

[17] Versura P, Cellini M, Torreggiani A (2001). The biocompatibility of silicone, fluorosilicone and perfluorocarbon liquids as vitreous tamponades. *Ophthalmologica*.

[18] Devin F, Jourdan T, Saracco JB, Lucciani A (1995). Experimental tolerance to perfluorodecalin used in prolonged intraocular tamponade. *Ophthalmologica*.

[19] Iwamoto T (1990). Histopathology of rabbit and pig retina in eyes with intravitreal perfluorochemicals, with special reference to pdd (photoreceptor drop down) and mep (moath eaten phenomenon). *Nippon Ganka Gakkai Zasshi*.

[20] Mathis A (1992). Experimental tolerance of perfluorodecalin. *Journal of Vitreo Retina*.

[21] Sparrow JR, Matthews GP, Iwamoto T, Ross R, Gershbein A, Chang S (1993). Retinal tolerance to intravitreal perfluoroethylcyclohexane liquid in the rabbit. *Retina*.

[22] Peyman GA, Conway MD, Soike KF, Clark LC (1991). Long-term vitreous replacement in primates with intravitreal vitreon or vitreon plus silicone. *Ophthalmic Surgery*.

[23] Eckardt C, Havsteen B, Winter M (1992). Biochemical analysis of perfluorocarbon induced vitreous precipitates: a preliminary study. * Journal of Vitreoretinal Surgery*.

[24] Drury B, Bourke RD (2011). Short-term intraocular tamponade with perfluorocarbon heavy liquid. *British Journal of Ophthalmology*.

[25] Suk KK, Flynn HW (2011). Management options for submacular perfluorocarbon liquid. *Ophthalmic Surgery Lasers and Imaging*.

[26] Velikay M, Stolba U, Wedrich A, Li Y, Datlinger P, Binder S (1995). The effect of chemical stability and purification of perfluorocarbon liquids in experimental extended-term vitreous substitution. *Graefe’s Archive for Clinical and Experimental Ophthalmology*.

[27] Sirimaharaj M, Balachandran C, Chan WC (2005). Vitrectomy with short term postoperative tamponade using perfluorocarbon liquid for giant retinal tears. *British Journal of Ophthalmology*.

[28] Ventura MC, Melo C, Escarião P, Diniz JR, Leão AC (2007). Perfluoroctano liquido como tamponante vitreoretiniano de curta duraçao no pós-operatório de portadores de descolamento de retina por ruptura gigante. *Arquivos Brasileiros de Oftalmologia*.

[29] Bottoni F, Bailo G, Arpa P, Prussiani A, Monticelli M, de Molfetta V (1994). Management of giant retinal tears using perfluorodecalin as a postoperative short-term vitreoretinal tamponade: a long-term follow-up study. *Ophthalmic Surgery*.

[30] Rizzo S, Genovesi-Ebert F, Vento A, Cresti F, di Bartolo E, Belting C (2007). A new heavy silicone oil (HWS 46-3000) used as a prolonged internal tamponade agent in complicated vitreoretinal surgery: a pilot study. *Retina*.

[31] Boscia F, Furino C, Recchimurzo N, Besozzi G, Sborgia G, Sborgia C (2008). Oxane HD vs silicone oil and scleral buckle in retinal detachment with proliferative vitreoretinopathy and inferior retinal breaks. *Graefe’s Archive for Clinical and Experimental Ophthalmology*.

[32] Sandner D, Engelmann K (2006). First experiences with high-density silicone oil (densiron) as an intraocular tamponade in complex retinal detachment. *Graefe’s Archive for Clinical and Experimental Ophthalmology*.

[33] Sigler EJ, Randolph JC, Calzada JI, Charles S (2013). 25-gauge pars plana vitrectomy with medium-term postoperative perfluorotamponade for inferior retinal detachment. *Ophthalmic Surgery, Lasers and Imaging Retina*.

[34] Sigler EJ, Randolph JC, Calzada JI, Charles S (2013). Pars plana vitrectomy with medium-term postoperative perfluoro-n-octane for recurrent inferior retinal detachment complicated with advanced proliferative vitreoretinopathy. *Retina*.

[35] Sigler EJ, Randolph JC, Charles S (2014). Foreign body response within postoperative perfluoro-n-octane for retinal detachment repair. *Retina*.

[36] Rofail M, Lee LR (2005). Perfluoro-n-octane as a postoperative vitreoretinal tamponade in the management of giant retinal tears. *Retina*.

[37] Rush R, Sheth S, Surka S, Ho I, Gregory-Roberts J (2012). Postoperative perfluoro-n-octane tamponade for primary retinal detachment repair. *Retina*.

[38] Singh J, Ramaesh K, Wharton SB, Cormack G, Chawla HB (2001). Perfluorodecalin-induced intravitreal inflammation. *Retina*.

[39] Ajami B, Bennett JL, Krieger C, Tetzlaff W, Rossi FM (2007). Local self-renewal can sustain CNS microglia maintenance and function throughout adult life. *Nature Neuroscience*.

[40] Gerber JS, Mosser DM (2001). Reversing lipopolysaccharide toxicity by ligating the macrophage fe gamma receptors. *Journal of Immunology*.

[41] Xu S, Wang P, Wei K (2013). Cytoprotection of perfluorocarbon on pmvecs *in vitro*. *Inflammation*.

[42] Edwards JP, Zhang X, Frauwirth KA, Mosser DM (2006). Biochemical and functional characterization of three activated macrophage populations. *Journal of Leukocyte Biology*.

[43] Kirchhof B, Wong D, van Meurs J (2002). Use of perfluorohexyloctane as a long-term internal tamponade agent in complicated retinal detachment surgery. *American Journal of Ophthalmology*.

[44] Verma LK, Peyman GA, Wafapoor H, Greve MD, Millsap CM, Adile SL (1995). An analysis of posterior segment complications after vitrectomy using the perfluorocarbon perfluoroperhydrophenanthrene (vitreon). vitreon collaborative study. *Ophthalmic Surgery*.

[45] Kertes PJ, Wafapoor H, Peyman GA, Calixto N, Thompson H (1997). The management of giant retinal tears using perfluoroperhydrophenanthrene. A multicenter case series. vitreon collaborative study group. *Ophthalmology*.

[46] Cauchi P, Azuara-Blanco A, McKenzie J (2005). Corneal toxicity and inflammation secondary to retained perfluorodecalin. *American Journal of Ophthalmology*.

[47] Wilbanks GA, Apel AJ, Jolly SS, Devenyi RG, Rootman DS (1996). Perfluorodecalin corneal toxicity: five case reports. *Cornea*.

[48] Veckeneer MA, de Voogd S, Lindstedt EW, Menz DH, van Meurs JC (2008). An epidemic of sticky silicone oil at the Rotterdam Eye Hospital. Patient review and chemical analyses. *Graefe’s Archive for Clinical and Experimental Ophthalmology*.

[49] Romano MR, Vallejo-Garcia JL, Parmeggiani F, Vito R, Vinciguerra P (2014). Interaction between perfluorcarbon liquid and heavy silicone oil: risk factor for “Sticky Oil” formation. *Current Eye Research*.

